# Effect of body mass index on diabetogenesis factors at a fixed fasting plasma glucose level

**DOI:** 10.1371/journal.pone.0189115

**Published:** 2018-01-29

**Authors:** Jiunn-Diann Lin, Chun-Hsien Hsu, Chung-Ze Wu, An-Tsz Hsieh, Chang-Hsun Hsieh, Yao-Jen Liang, Yen-Lin Chen, Dee Pei, Jin-Biou Chang

**Affiliations:** 1 Division of Endocrinology, Department of Internal Medicine, Shuang Ho Hospital, Division of Endocrinology and Metabolism, School of Medicine, College of Medicine, Taipei Medical University, Taipei, Taiwan, ROC; 2 Department of Family Medicine, Cardinal Tien Hospital, Fu Jen Catholic University School of Medicine, New Taipei City, Taiwan, ROC; 3 Division of Endocrinology and Metabolism, Department of Internal Medicine, Tri-Service General Hospital, National Defense Medical School, Taipei, Taiwan, ROC; 4 Department of Life Science, Fu Jen Catholic University, New Taipei City, Taiwan, ROC; 5 Department of Pathology, Cardinal Tien Hospital, Fu Jen Catholic University School of Medicine, New Taipei City, Taiwan, ROC; 6 Department of Internal Medicine, Cardinal Tien Hospital, Fu Jen Catholic University, New Taipei City, Taiwan, ROC; 7 Department of Pathology, National Defense Medical Center, Division of Clinical Pathology, Tri-Service General Hospital, Taipei, Taiwan, ROC; University of Rochester, UNITED STATES

## Abstract

**Aim:**

The present study evaluated the relative influence of body mass index (BMI) on insulin resistance (IR), first-phase insulin secretion (FPIS), second-phase insulin secretion (SPIS), and glucose effectiveness (GE) at a fixed fasting plasma glucose level in an older ethnic Chinese population.

**Methods:**

In total, 265 individuals aged 60 years with a fasting plasma glucose level of 5.56 mmol/L were enrolled. Participants had BMIs of 20.0–34.2 kg/m^2^. IR, FPIS, SPIS, and GE were estimated using our previously developed equations. Pearson correlation analysis was conducted to assess the correlations between the four diabetogenesis factors and BMI. A general linear model was used to determine the differences in the percentage of change among the four factor slopes against BMI.

**Results:**

Significant correlations were observed between BMI and FPIS, SPIS, IR, and GE in both women and men, which were higher than those reported previously. In men, BMI had the most profound effect on SPIS, followed by IR, FPIS, and GE, whereas in women, the order was slightly different: IR, followed by FPIS, SPIS, and GE. Significant differences were observed among all these slopes, except for the slopes between FPIS and SPIS in women (p = 0.856) and IR and FPIS in men (p = 0.258).

**Conclusions:**

The contribution of obesity to all diabetes factors, except GE, was higher than that reported previously. BMI had the most profound effect on insulin secretion in men and on IR in women in this 60-year-old cohort, suggesting that lifestyle modifications for obesity reduction in women remain the most important method for improving glucose metabolism and preventing future type 2 diabetes mellitus.

## Introduction

Increased insulin resistance (IR) and deteriorated insulin secretion (ISEC) are considered to be the main mechanisms in type 2 diabetes mellitus (T2DM) development [1]. Evidence has indicated that increased β-cell function maintains the glucose equilibrium in individuals with increased IR [2]. However, overt diabetes eventually develops after the failure of β-cell secretion compensation [1, 2]. Even in the stage of clinically evident diabetes, satisfactory glycemic control can be maintained through lifestyle modifications and medication use, which can improve both IR and β-cell function. However, ISEC consists of two phases: first-phase insulin secretion (FPIS) and second-phase insulin secretion (SPIS) [3, 4]. No direct evidence has supported the aforementioned observation because FPIS disappears early in the prediabetes stage; therefore, SPIS must be responsible for glucose control under oral medications [5].

Glucose clearance from the circulatory system to the muscles, liver, and fat tissues occurs through two pathways: insulin- and non-insulin-mediated glucose uptake. Non-insulin-mediated glucose uptake is also referred to as glucose effectiveness (GE), which represents the ability of glucose to increase its own cellular uptake and restrain its endogenous hepatic output under basal insulin levels. Best *et al*. reported that GE accounts for 66% of glucose metabolism in healthy individuals but provides 99% of glucose metabolism in patients with T2DM due to high IR and severe insulin deficiency [6]. Therefore, the deterioration of GE has been argued to play a significant role in the occurrence of T2DM [7]. However, very few studies to date have focused on the importance of GE [6]. Therefore, in this study we proposed that IR, GE, FPIS, and SPIS are the four most important factors for diabetes development and control, referred to as diabetogenesis factors (DFs).

Obesity is positively related to high IR and contributes to high β-cell function [8–10]. This increased β-cell mass and resulting increased ISEC might be because of the compensatory reaction to high IR [11, 12]. This aspect of diabetes pathophysiology has been studied quite extensively. However, the effects of obesity on GE or different ISEC stages remain undetermined. For example, Lopez *et al*. reported that GE deteriorates as body mass index (BMI) increases in nondiabetic individuals, whereas Healy *et al*. reported contrasting findings [13, 14].

The national health insurance policy in Taiwan has caused a continuous increase in the average life expectancy. Officially, Taiwan became an aging society in 2014, with 11.7% of the population aged more than 65 years [15], which has resulted in a simultaneous rise in T2DM prevalence. Therefore, understanding T2DM pathophysiology is increasingly important.

In the present study, IR, FPIS, SPIS, and GE were examined in the same individuals to evaluate the effects of BMI on the four DFs in the older population. Age and blood glucose levels also affect these four DFs; therefore, we only enrolled individuals with the same age (60 years) and fasting plasma glucose (FPG) level (5.56 mmol/L) to investigate the actual relationships.

## Materials and methods

### 2.1. Ethics

The subjects of the current study were enrolled form the data bank of Meei-Jaw (MJ) Health Screening Centers and Cardinal Tien Hospital data access center between 1999 and 2008. All study subjects were anonymous, and informed consent was obtained prior to participation. These data do not contain potentially identifying or sensitive patient information, data are not owned by a third-party organization. The study proposal was reviewed and approved by the institutional review board of MJ Health Screening Center joint of Cardinal Tien Hospital before the study began. The contact information for the Cardinal Tien Hospital Data Access Committee is +88622219331.

### 2.2. Participants

The data on the individuals enrolled in the current study were obtained from the databank of the Meei-Jaw (MJ) Health Screening Center for 1999–2008. All study participants remained anonymous, and informed consent was obtained prior to participation. The study proposal was reviewed and approved by the Institutional Review Board of MJ Health Screening Center before the study began. In total, 265 individuals with the same FPG level (5.56 mmol/L) and age (60 years) were enrolled to eliminate the profound effects of age and glucose metabolism. Under these criteria, BMI ranged from 20.0 to 34.2 kg/m^2^. The participants had no other significant diseases, no history of diabetes or diabetic ketoacidosis, and did not use any medication known to influence insulin sensitivity or β-cell function (including oral antihyperglycemic agents) during the study period. BMI was calculated as body weight (kg)/height (m^2^). Waist circumference (WC) was measured horizontally at the level of the natural waist, which was identified as the level at the hollow molding of a laterally concave trunk. Systolic blood pressure (SBP) and diastolic blood pressure (DBP) were measured in the right arm of seated individuals by using a standard mercury sphygmomanometer. Blood samples were drawn from the antecubital vein for biochemical analysis.

### 2.3. Calculations of IR, FPIS, SPIS, and GE

IR, FPIS, SPIS, and GE were estimated using our previously developed equations, listed as follows:

IR: (1.439 + 0.018 × sex– 0.003 × age + 0.029 BMI– 0.001 × SBP + 0.006 DBP + 0.049 × TG– 0.046 × HDL-C– 0.016 × FPG) × 10^3.333^ [16];
log (FPIS) = 1.477–0.119 × FPG + 0.079 × BMI– 0.523 × HDL-C [17];
log (SPIS) = -2.400–0.088 × FPG + 0.072 × BMI [18]; and
GE = (29.196 − 0.103 × age − 2.722 × TG − 0.592 × FPG) × 10^−3^[19], where HDL-C and TG represent high-density lipoprotein cholesterol and triglycerides, respectively.

### 2.4. Laboratory evaluation

After 10-hour overnight fasting, blood samples were collected from each individual for further analysis. The plasma was separated from the whole blood within 1 hour and stored at −70°C. FPG and plasma lipid levels were measured subsequently. The glucose oxidase method (YSI 203 glucose analyzer; Scientific Division, Yellow Springs Instruments, Yellow Springs, OH, USA) was used to determine FPG levels. The dry, multilayer analytical slide method with the Fuji Dri-Chem 3000 analyzer (Fuji Photo Film, Minato-Ku, Tokyo, Japan) was used to determine total cholesterol and triglyceride (TG) levels. An enzymatic cholesterol assay following dextran sulfate precipitation was used to determine serum HDL-C and low-density lipoprotein cholesterol (LDL-C) levels.

### 2.5. Statistical analysis

Data are represented as means ± standard deviations. The Student t test was used to evaluate the differences of continuous data between men and women. One-way analysis of variance was used to assess differences in the demographic data, clinical parameters, and DFs among the normal, overweight, and obese groups. The Bonferroni test was used for post hoc analysis. The correlations between the BMI and DFs were evaluated by Pearson correlation analysis. Because the units and scales for these four lines were different, it was difficult to construct all four slopes in one figure. To resolve this problem, we transformed the absolute units into relative units, which represent the percentage of changes in that particular DF, rather than plotting each parameter against the BMI using the original units (e.g., μU/min for FPIS). For example, the lowest and highest values of FPIS (6.06 and 899.10 μU/min, respectively) were regarded as 0% and 100%, respectively. Notably, this transformation did not change the r values of each slope. Other FPIS values were calculated using the following equation:

Percentage of changes in the FPIS value = [(899.1 –the value)/(899.1–6.06)]/100

Similar methods were used to assess the slopes between BMI and IR, GE, and SPIS to compare the changes in the data across the same range of FPG levels. A general linear model was used to determine the differences among the four slopes against the BMI. All statistical tests were two-sided, and p < 0.05 was considered statistically significant. Statistical analysis was performed using SPSS 10.0 for Windows (SPSS, Chicago, IL, USA).

## Results

In total, 122 men and 143 women were enrolled in this study. Table 1 presents the demographic characteristics, biochemical data, and DFs. Notably, men had higher WC and higher FPIS but lower HDL-C levels than women.

**Table 1:** Demographic characteristics and indices of glucose metabolism in all participants with or without metabolic syndrome.

	Women			Men			p value
N	143			122			
Body mass index (kg/m^2^)	23.9	±	2.5	23.9	±	2.3	1.000
Waist circumference (cm)	75.7	±	6.7	84.1	±	7.8	<0.001
Body fat (%)	32.3	±	4.8	22.4	±	4.6	<0.001
SBP (mmHg)	128.6	±	21.6	126.1	±	16.9	0.516
DBP (mmHg)	75.0	±	12.0	76.6	±	11.7	0.443
Triglyceride (mmol/L)	1.4	±	0.8	1.4	±	0.7	0.503
HDL-cholesterol (mmol/L)	1.6	±	0.3	1.3	±	0.3	<0.001
Total cholesterol (mmol/L)	5.6	±	0.9	5.2	±	1.0	0.002
FPIS (μU/min)	98.9	±	82.1	128.4	±	85.4	0.004
SPIS (pmol/mmol)	0.075	±	0.041	0.074	±	0.033	0.803
IR (10^−4^ min^−1^ pmol^−1^ L^−1^)	3.68	±	0.02	3.69	±	0.02	0.535
GE (10^−2^ dL min^−1^ kg^−1^)	0.016	±	0.002	0.015	±	0.0022	0.509

SBP, systolic blood pressure; DBP, diastolic blood pressure; HDL, high density lipoprotien; FPIS, first phase insulin secretion; SPIS, second phase insulin secretion; IR, insulin resistance; GE, glucose effectiveness. Data are presented as mean ± SD

Table 2 shows the changes in the studied parameters in normal, overweight, and obese groups. The obese group had the highest WC, IR, FPIS, and SPIS. The overweight group had higher WC, FPIS, SPIS, and IR than the normal group. However, obese women had higher TG but lower HDL-C levels and GE than normal women.

**Table 2:** Comparison of the demographic characteristics and indices of glucose metabolism in different groups.

	Normal group			Overweight group			Obese group		
Women									
N	80			45			18		
BMI (kg/m^2^)	22.2	±	1.2^2,3^	25.3	±	0.8^1,3^	28.5	±	1.8^1,2^
Waist circumference (cm)	72.2	±	4.7^2,3^	77.6	±	4.1^1,3^	86.5	±	6.2^1,2^
SBP (mmHg)	126.5	±	21.0	129.2	±	19.7	131.1	±	16.1
DBP (mmHg)	74.2	±	12.4	76.2	±	12.1	75.6	±	10.5
Triglyceride (mmol/L)	1.2	±	0.7^3^	1.5	±	0.8	1.7	±	0.8^1^
HDL-cholesterol (mmol/L)	1.6	±	0.3^3^	1.6	±	0.4	1.3	±	0.2^1^
Total cholesterol (mmol/L)	5.6	±	0.9	5.6	±	0.7	5.5	±	1.1
FPIS (μU/min)	58.6	±	27.1^2,3^	106.8	±	5 0.6^1,3^	257.9	±	108.2^1,2^
SPIS (pmol/mmol)	0.052	±	0.010^2,3^	0.086	±	0.012^1,3^	0.153	±	0.061^1,2^
IR (10^−4^ min^−1^ pmol^−1^ L^−1^)	3.67	±	0.02^2,3^	3.69	±	0.02^1,3^	3.71	±	0.01^1,2^
GE (10^−2^ dL min^−1^ kg^−1^)	0.016	±	0.002^3^	0.016	±	0.002	0.015	±	0.002^1^
Men									
N	64			44			14		
BMI (kg/m^2^)	22.2	±	1.1^2,3^	25.1	±	0.8^1,3^	28.5	±	1.2^1,2^
Waist circumference (cm)	79.2	±	4.9^2,3^	87.3	±	5.5^1,3^	96.5	±	5.7^1,2^
SBP (mmHg)	123.4	±	15.8	130.3	±	17.9	125.9	±	17.1
DBP (mmHg)	74.9	±	10.5	79.3	±	13.3	76.2	±	10.5
Triglyceride (mmol/L)	1.3	±	0.7	1.6	±	0.7	1.4	±	0.6
HDL-cholesterol (mmol/L)	1.3	±	0.3	1.2	±	0.4	1.3	±	0.3
Total cholesterol (mmol/L)	5.2	±	1.0	5.2	±	0.9	5.6	±	1.1
FPIS (μU/min)	79.1	±	30.4^2,3^	153.2	±	60.8^1,3^	257.9	±	117.0^1,2^
SPIS (pmol/mmol)	0.052	±	0.009^2,3^	0.083	±	0.011^1,3^	0.148	±	0.030^1,2^
IR (10^−4^ min^−1^ pmol^−1^ L^−1^)	3.67	±	0.02^2,3^	3.69	±	0.01^1,3^	3.71	±	0.01^1,2^
GE (10^−2^ dL min^−1^ kg^−1^)	0.016	±	0.002	0.015	±	0.002	0.016	±	0.002

Normal = 18.5 ≤ BMI < 24; overweight = 24 ≤ BMI < 27; obese = BMI ≥ 27. BMI = body mass index; HDL = high-density lipoprotein; FPIS = first-phase insulin secretion; SPIS = second-phase insulin secretion; IR = insulin resistance; GE = glucose effectiveness. Data are presented as means ± SDs. 1 *p*< 0.05 to normal group; 2*p*< 0.05 to overweight group; 3*p*< 0.05 to obese group.

**Fig 1:**
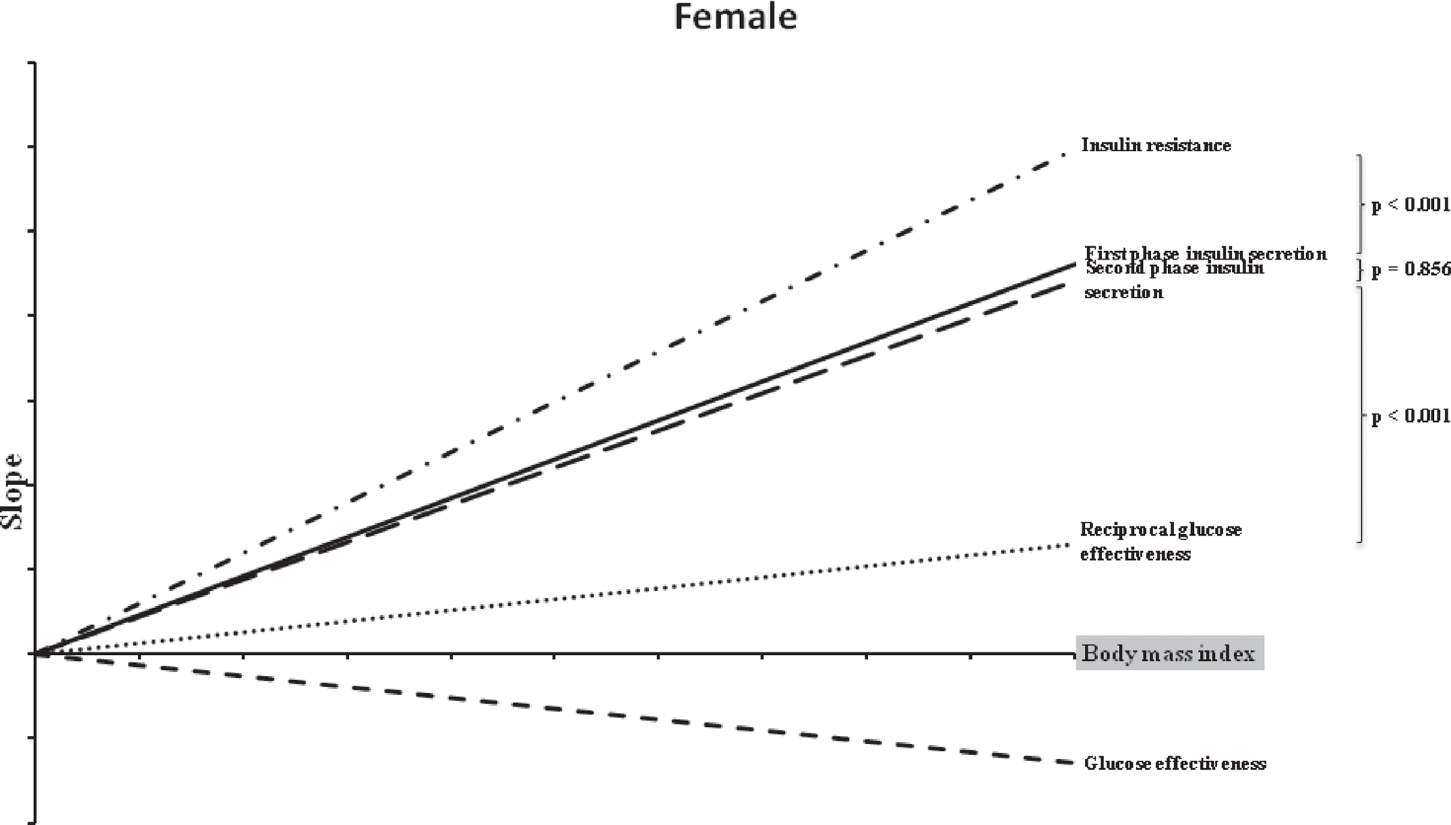
Slopes of insulin resistance, first-phase and second-phase insulin secretions, and glucose effectiveness against the body mass indices of women.

**Fig 2:**
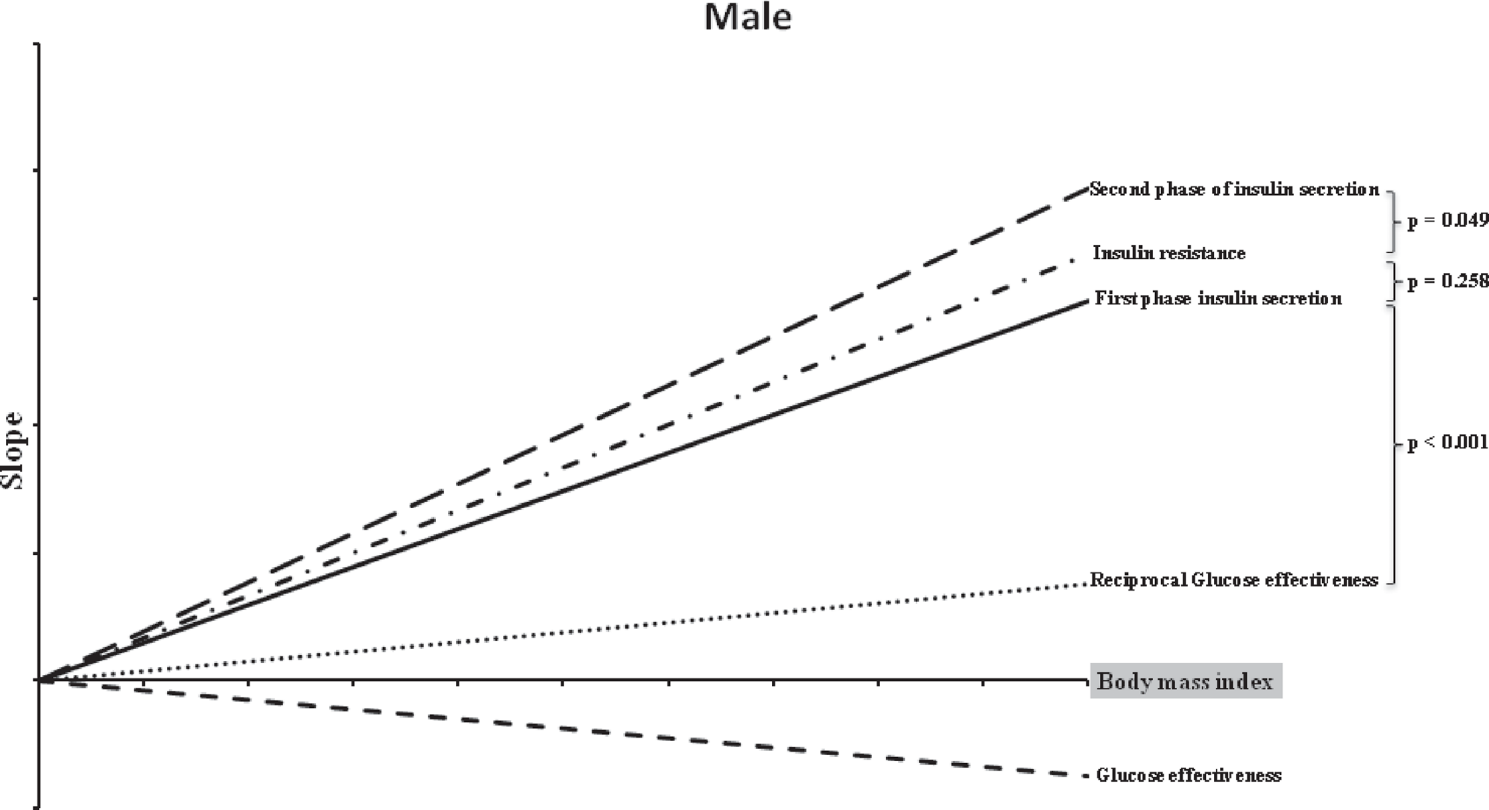
Slopes of insulin resistance, first-phase and second-phase insulin secretions, and glucose effectiveness against the body mass indices of men.

Table 3 presents the correlations between the BMI and DFs. These correlations were significant in both men and women. Fig 1 and Fig 2 present the most important result of the present study: the comparison of the slopes of the BMI and the four DFs in women and men, respectively. As mentioned previously, these slopes were obtained by transforming the absolute units into relative units (% changes) before comparison. In men, BMI had the most profound effect on SPIS, followed by IR, FPIS, and GE, whereas in women, the order was slightly different: IR, followed by FPIS, SPIS, and GE. However, a significant difference was observed between all these slopes, except for the slopes between FPIS and SPIS in women (p = 0.856) and IR and FPIS in men (p = 0.258). The relevant data (1061012-MJ-BMI) is shown in S1 File. All relevant results are within the paper and its Supporting Information files.

**Table 3:** The correlations between the body mass index and diabetes factors.

	FPIS	SPIS	IR	GE
Women	0.813	0.926	0.730	-0.203
Men	0.795	0.966	0.780	-0.204

FPIS = first-phase insulin secretion; SPIS = second-phase insulin secretion; IR = insulin resistance; and GE = glucose effectiveness. Data are presented as r values; all p < 0.001.

## Discussion

Age and FPG levels affect all DFs; therefore, they were included as confounding factors. The present study specifically enrolled individuals with the same age (60 years) and FPG level (5.56 mmol/L) to avoid these effects. Therefore, we evaluated the pure effect of BMI on these four DFs. Our results demonstrate that IR, FPIS, and SPIS are highly correlated with BMI compared with the findings of previous studies, suggesting that the contribution of obesity to FPIS, SPIS, and IR is more important than generally expected. In addition, the present results demonstrate that the contributions of the four DFs differ between women and men. In women, IR was most strongly correlated with BMI, followed by FPIS, SPIS, and GE. However, the order changed to SPIS, IR, FPIS, and GE in men. Our results not only improve understanding of T2DM pathophysiology but also can be applied in the clinical prevention and management of T2DM.

The study individuals with the same FPG level were categorized into normal, overweight, and obese groups. In addition, the clinical parameters and DFs were compared among the three groups. The results indicate that obesity has a significant influence on TG and HDL-C levels in women but not in men, which can be attributed to the differences in the basic sex hormone effects on the regulation of lipid metabolism [20, 21] and the diversity in insulin-mediated free fatty acid and TG metabolisms between women and men [22]. Furthermore, FPIS, SPIS, and IR increased markedly from the normal to overweight groups and further increased from the overweight to obese groups (Table 2). However, GE did not change significantly in men and decreased slightly from normal to obese women, indicating that the pure effect of BMI on GE is minor in both women and men (Table 1).

Increasing evidence has shown that obesity is the core contributor to IR [23–25]. BMI reduction through lifestyle modifications can improve IR, which could reduce the incidence of T2DM [23–25]. For the established T2DM individuals, improved IR is equivalent to appropriate glucose control [23–25]. The relationship between BMI and IR has been studied extensively in different ethnic groups. However, the range of r values was approximately 0.4–0.59 [9, 26–28]. In the present study, the r value obtained using our previously derived equations was the highest (r = 0.747, Table 3) compared with other studies [16]. This minor discrepancy in results might be due to differences in genetic background, sample size, glucose levels, and inclusion criteria, as well as BMI. The BMIs reported in previous studies are higher than that reported in the present study (27–28 kg/m^2^ vs. 24.2 kg/m^2^). Notably, no previous studies have used a cohort with the same age and FPG level. We believed that by fixing the range of these two confounders, the true effect of BMI could be elucidated.

Obese individuals have higher β-cell function because they have higher cell mass than lean individuals [27,29,30]. Hanley *et al*. conducted the frequently sampled intravenous glucose tolerance test (FSIGT) for FPIS measurement and reported that FPIS is only weakly associated with BMI (r = 0.14) in nondiabetic individuals (age: 53–54 years, BMI: 28–30) [27]. Another study used clamps to measure SPIS and revealed the same finding that BMI is correlated with both FPIS and SPIS (r = 0.28 and 0.41, respectively) in Caucasians without diabetes [28]. However, this study was conducted on a younger cohort (BMI: 26–27, age 42–48 years). In accordance with these studies, our findings demonstrate that FPIS is strongly correlated with BMI (r = 0.796, p < 0.001).

The importance of SPIS remains unclear. The relative importance of ISEC and IR differs between Caucasians and Asians [31]. For many Asian patients with diabetes, glucose can be controlled satisfactorily for many years through oral hypoglycemic drugs. Because FPIS disappears early in the prediabetes stage, it is reasonable to postulate that SPIS must be the most important cause of glucose control before insulin injection. The present study successfully demonstrates that, similar to FPIS, SPIS is correlated with BMI (r = 0.949, p < 0.001). This relationship can be easily explained by the compensation ability of β-cells in response to increasing IR [8, 9, 30, 32]. Notably, the r values in this study were also higher than those reported in previous studies (0.01–0.1). Furthermore, old age, a highly homogeneous study cohort, and a lower BMI could have contributed to this discrepancy in results. Therefore, additional studies are warranted to confirm our findings [9, 27, 30].

Although the effect of BMI on GE has been demonstrated in several studies, the results remain controversial. In the present study, GE was negatively correlated with BMI (r = −0.232) which is consistent with the results reported by Kautzky-Willer *et al*. and Lopes *et al*. [13, 33] but not with those reported by Healy *et al*. [14]. By conducting the FSIGT for measuring GE in white and African Americans, Healy *et al*. showed that no correlation was present in individuals with prediabetes. Plausible explanations for these inconsistent results include different ethnic populations, inclusion criteria, GE estimation methods, and the BMI (37.8 ± 6.3 kg/m^2^) and young age (46.5 ± 11.2 years). In particularly, very few Chinese individuals have a comparable BMI.

IR is likely the key factor explaining the relationship between BMI and GE. According to the aforementioned discussion, the positive correlation between BMI and IR is confirmed [27]. Furthermore, the results of our studies and Lopez *et al*. all indicate that IR is negatively correlated with GE (r = −0.462, p < 0.001, and r = −0.69, p < 0.001, respectively) [13]. Therefore, through IR, BMI is associated with GE. From a physiological perspective, substantial evidence also supports our results. Obese individuals have higher IR and serum free fatty acids [34, 35]. Hawkins *et al*. showed that increased free fatty acid levels can deteriorate GE in T2DM [34, 35].

The risk, pathophysiology, and complications of T2DM differ between men and women [36]. For instance, compared with men, women with diabetes tend to be older and have a higher BMI [36–38]. The present study also investigated the differences in the aforementioned relationships between men and women. Our initial analysis results demonstrate that both men and women have similar r values derived from a simple correlation (Table 3). To further determine the relative contributions of BMI to the DFs, we transformed the absolute units to relative units (% changes) to compare the four slopes. Notably, after the transformation, the relationships became different. In women, BMI has the most profound influence on IR, followed by FPIS, SPIS, and GE. In men, the order changed to SPIS, IR, FPIS, and GE. These observations were comparable with those reported in the study by Kautzky-Willer *et al*, in which the association of BMI with β-cell function was sex-biased [39]. They revealed that although the slopes of IR and BMI were similar in both men and women, ISEC had a steeper increase in men than in women, indicating that men have a higher compensation capacity in response to IR than women [39]. This phenomenon can also be explained by differences in sex hormones, body fat composition, body fat distribution, and adipocytokines [36, 39].

The current study has some limitations. First, this is a cross-sectional study. Therefore, our data are less persuasive than those of a longitudinal study. Second, family history of T2DM, which is also a key factor for future diabetes development, was not evaluated in our established equations. The inclusion of this factor in the equations might increase the accuracy of our factor measurements. Third, only Chinese individuals were enrolled in our study; therefore, our study results should be applied with caution to other ethnic groups.

In conclusion, in men, BMI has the most profound effect on ISEC; however, in women, IR is the most important DF. Although GE is least correlated with BMI, it is still significant. Therefore, the role of GE should not be overlooked in this homogeneous cohort with the same age and FPG level.

## Supporting information

S1 File (XLS)

## Abbreviations

SI: insulin sensitivity
FPIS: first-phase insulin secretion
SPIS: second-phase insulin secretion
IR: insulin resistance
FPG: fasting plasma glucose
T2DM: type 2 diabetes mellitus
BMI: body mass index
WC: waist circumference

## References

[1] DeFronzo RA, Tobin JD, Andres R (1979) Glucose clamp technique: a method for quantifying insulin secretion and resistance. American Journal of Physiology 237: E214–223. doi: 10.1152/ajpendo.1979.237.3.E214 382871 10.1152/ajpendo.1979.237.3.E214

[2] Kahn SE (2003) The relative contributions of insulin resistance and beta-cell dysfunction to the pathophysiology of Type 2 diabetes. Diabetologia 46: 3–19. doi: 10.1007/s00125-002-1009-0 12637977 10.1007/s00125-002-1009-0

[3] Polonsky KS, Sturis J, Bell GI (1996) Seminars in Medicine of the Beth Israel Hospital, Boston. Non-insulin-dependent diabetes mellitus—a genetically programmed failure of the beta cell to compensate for insulin resistance. The New England Journal of Medicine 334: 777–783. doi: 10.1056/NEJM199603213341207 8592553 10.1056/NEJM199603213341207

[4] Caumo A, Luzi L (2004) First-phase insulin secretion: does it exist in real life? Considerations on shape and function. Am J Physiol Endocrinol Metab 287: E371–385. doi: 10.1152/ajpendo.00139.2003 15308473 10.1152/ajpendo.00139.2003

[5] Pratley RE, Weyer C (2001) The role of impaired early insulin secretion in the pathogenesis of Type II diabetes mellitus. Diabetologia 44: 929–945. doi: 10.1007/s001250100580 11484070 10.1007/s001250100580

[6] Best JD, Kahn SE, Ader M, Watanabe RM, Ni TC, Bergman RN (1996) Role of glucose effectiveness in the determination of glucose tolerance. Diabetes Care 19: 1018–1030. 8875104 10.2337/diacare.19.9.1018

[7] Basu A, Caumo A, Bettini F, Gelisio A, Alzaid A, Cobelli C, et al (1997) Impaired basal glucose effectiveness in NIDDM: contribution of defects in glucose disappearance and production, measured using an optimized minimal model independent protocol. Diabetes 46: 421–432. 9032098 10.2337/diab.46.3.421

[8] Chiu KC, Cohan P, Lee NP, Chuang LM (2000) Insulin sensitivity differs among ethnic groups with a compensatory response in beta-cell function. Diabetes Care 23: 1353–1358. 10977032 10.2337/diacare.23.9.1353

[9] Chiu KC, Chuang LM, Yoon C (2001) Comparison of measured and estimated indices of insulin sensitivity and beta cell function: impact of ethnicity on insulin sensitivity and beta cell function in glucose-tolerant and normotensive subjects. J Clin Endocrinol Metab 86: 1620–1625. doi: 10.1210/jcem.86.4.7432 11297594 10.1210/jcem.86.4.7432

[10] Lin JD, Chang JB, Wu CZ, Pei D, Hsieh CH, Hsieh AT, et al (2014) Identification of insulin resistance in subjects with normal glucose tolerance. Ann Acad Med Singapore 43: 113–119. 24652432

[11] Schuster DP, Kien CL, Osei K (1998) Differential impact of obesity on glucose metabolism in black and white American adolescents. Am J Med Sci 316: 361–367. 9856689 10.1097/00000441-199812000-00002

[12] Polonsky KS, Given BD, Van Cauter E (1988) Twenty-four-hour profiles and pulsatile patterns of insulin secretion in normal and obese subjects. J Clin Invest 81: 442–448. doi: 10.1172/JCI113339 3276730 10.1172/JCI113339 PMC329589

[13] Lopez X, Bouche C, Tatro E, Goldfine AB (2009) Family history of diabetes impacts on interactions between minimal model estimates of insulin sensitivity and glucose effectiveness. Diabetes Obes Metab 11: 123–130. doi: 10.1111/j.1463-1326.2008.00913.x 18518893 10.1111/j.1463-1326.2008.00913.x

[14] Healy SJ, Osei K, Gaillard T (2015) comparative study of glucose homeostasis, lipids and lipoproteins, HDL functionality, and cardiometabolic parameters in modestly severely obese African Americans and White Americans with prediabetes: implications for the metabolic paradoxes. Diabetes Care 38: 228–235. doi: 10.2337/dc14-1803 25524949 10.2337/dc14-1803 PMC4302264

[15] Wang SL, Pan WH, Hwu CM, Ho LT, Lo CH, Lin SL, et al (1997) Incidence of NIDDM and the effects of gender, obesity and hyperinsulinaemia in Taiwan. Diabetologia 40: 1431–1438. doi: 10.1007/s001250050846 9447951 10.1007/s001250050846

[16] Wu CZ, Lin JD, Hsia TL, Hsu CH, Hsieh CH, Chang JB, et al (2014) Accurate method to estimate insulin resistance from multiple regression models using data of metabolic syndrome and oral glucose tolerance test. J Diabetes Investig 5: 290–296. doi: 10.1111/jdi.12155 24843777 10.1111/jdi.12155 PMC4020333

[17] Lin JD, Hsu CH, Liang YJ, Lian WC, Hsieh CH, Wu CZ, et al (2015) The estimation of first-phase insulin secretion by using components of the metabolic syndrome in a chinese population. Int J Endocrinol 2015: 675245 doi: 10.1155/2015/675245 25815010 10.1155/2015/675245 PMC4359803

[18] Lin YT, Wu CZ, Lian W.C., Hsu CH, Hsieh CH, Pei D, et al (2015) Measuring second phase of insulin secretion by components of metabolic syndrome. International Journal of Diabetes and Clinical Diagnosis 2: 113–118.

[19] Chen YL, Lee SF, Pei C, Pei D, Lee CH, He CT, et al (2016) Predicting Glucose Effectiveness in Chinese Participants Using Routine Measurements. Metab Syndr Relat Disord 14: 386–390. doi: 10.1089/met.2015.0136 27461066 10.1089/met.2015.0136

[20] D'Eon TM, Souza SC, Aronovitz M, Obin MS, Fried SK, Greenberg AS (2005) Estrogen regulation of adiposity and fuel partitioning. Evidence of genomic and non-genomic regulation of lipogenic and oxidative pathways. J Biol Chem 280: 35983–35991. doi: 10.1074/jbc.M507339200 16109719 10.1074/jbc.M507339200

[21] Gao H, Bryzgalova G, Hedman E, Khan A, Efendic S, Gustafsson JA, et al (2006) Long-term administration of estradiol decreases expression of hepatic lipogenic genes and improves insulin sensitivity in ob/ob mice: a possible mechanism is through direct regulation of signal transducer and activator of transcription 3. Mol Endocrinol 20: 1287–1299. doi: 10.1210/me.2006-0012 16627594 10.1210/me.2006-0012

[22] McKeigue PM, Laws A, Chen YD, Marmot MG, Reaven GM (1993) Relation of plasma triglyceride and apoB levels to insulin-mediated suppression of nonesterified fatty acids. Possible explanation for sex differences in lipoprotein pattern. Arterioscler Thromb 13: 1187–1192. 8343493 10.1161/01.atv.13.8.1187

[23] Bloomgarden ZT (1998) Insulin resistance: current concepts. Clin Ther 20: 216–231; discussion 215. 9589814 10.1016/s0149-2918(98)80086-6

[24] Vega GL (2001) Results of Expert Meetings: Obesity and Cardiovascular Disease. Obesity, the metabolic syndrome, and cardiovascular disease. Am Heart J 142: 1108–1116. 11717620 10.1067/mhj.2001.119790

[25] (2004) Body mass index and insulin resistance. Obstet Gynecol 104: 5S–10S. doi: 10.1097/01.AOG.0000138805.07080.5e 15458928 10.1097/01.AOG.0000138805.07080.5e

[26] Cheal KL, Abbasi F, Lamendola C, McLaughlin T, Reaven GM, Ford ES (2004) Relationship to insulin resistance of the adult treatment panel III diagnostic criteria for identification of the metabolic syndrome. Diabetes 53: 1195–1200. 15111486 10.2337/diabetes.53.5.1195

[27] Hanley AJ, Wagenknecht LE, D'Agostino RB Jr., Zinman B, Haffner SM (2003) Identification of subjects with insulin resistance and beta-cell dysfunction using alternative definitions of the metabolic syndrome. Diabetes 52: 2740–2747. 14578292 10.2337/diabetes.52.11.2740

[28] van Haeften TW, Pimenta W, Mitrakou A, Korytkowski M, Jenssen T, Yki-Jarvinen H, et al (2000) Relative conributions of beta-cell function and tissue insulin sensitivity to fasting and postglucose-load glycemia. Metabolism 49: 1318–1325. 11079822 10.1053/meta.2000.9526

[29] Kloppel G, Lohr M, Habich K, Oberholzer M, Heitz PU (1985) Islet pathology and the pathogenesis of type 1 and type 2 diabetes mellitus revisited. Survey and Synthesis of Pathology Research 4: 110–125. 3901180 10.1159/000156969

[30] van Haeften TW, Dubbeldam S, Zonderland ML, Erkelens DW (1998) Insulin secretion in normal glucose-tolerant relatives of type 2 diabetic subjects. Assessments using hyperglycemic glucose clamps and oral glucose tolerance tests. Diabetes Care 21: 278–282. 9539996 10.2337/diacare.21.2.278

[31] Moller JB, Dalla Man C, Overgaard RV, Ingwersen SH, Tornoe CW, Pedersen M, et al (2014) Ethnic differences in insulin sensitivity, beta-cell function, and hepatic extraction between Japanese and Caucasians: a minimal model analysis. J Clin Endocrinol Metab 99: 4273–4280. doi: 10.1210/jc.2014-1724 25119313 10.1210/jc.2014-1724

[32] van Haeften TW, Pimenta W, Mitrakou A, Korytkowski M, Jenssen T, Yki-Jarvinen H, et al (2002) Disturbances in beta-cell function in impaired fasting glycemia. Diabetes 51 Suppl 1: S265–270. 11815491 10.2337/diabetes.51.2007.s265

[33] Kautzky-Willer A, Pacini G, Ludvik B, Schernthaner G, Prager R (1992) Beta-cell hypersecretion and not reduced hepatic insulin extraction is the main cause of hyperinsulinemia in obese nondiabetic subjects. Metabolism 41: 1304–1312. 1461136 10.1016/0026-0495(92)90100-o

[34] Roden M, Price TB, Perseghin G, Petersen KF, Rothman DL, Cline GW, et al (1996) Mechanism of free fatty acid-induced insulin resistance in humans. J Clin Invest 97: 2859–2865. doi: 10.1172/JCI118742 8675698 10.1172/JCI118742 PMC507380

[35] Hawkins M, Tonelli J, Kishore P, Stein D, Ragucci E, Gitig A, et al (2003) Contribution of elevated free fatty acid levels to the lack of glucose effectiveness in type 2 diabetes. Diabetes 52: 2748–2758. 14578293 10.2337/diabetes.52.11.2748

[36] Kautzky-Willer A, Harreiter J, Pacini G (2016) Sex and Gender Differences in Risk, Pathophysiology and Complications of Type 2 Diabetes Mellitus. Endocr Rev 37: 278–316. doi: 10.1210/er.2015-1137 27159875 10.1210/er.2015-1137 PMC4890267

[37] Wandell PE, Carlsson AC (2014) Gender differences and time trends in incidence and prevalence of type 2 diabetes in Sweden—a model explaining the diabetes epidemic worldwide today? Diabetes Res Clin Pract 106: e90–92. doi: 10.1016/j.diabres.2014.09.013 25451899 10.1016/j.diabres.2014.09.013

[38] Bray GA (2004) Medical consequences of obesity. J Clin Endocrinol Metab 89: 2583–2589. doi: 10.1210/jc.2004-0535 15181027 10.1210/jc.2004-0535

[39] Kautzky-Willer A, Brazzale AR, Moro E, Vrbikova J, Bendlova B, Sbrignadello S, et al (2012) Influence of increasing BMI on insulin sensitivity and secretion in normotolerant men and women of a wide age span. Obesity (Silver Spring) 20: 1966–1973. 22282046 10.1038/oby.2011.384

