# Correction: Effect of body mass index on diabetogenesis factors at a fixed fasting plasma glucose level

**DOI:** 10.1371/journal.pone.0197212

**Published:** 2018-05-07

**Authors:** Jiunn-Diann Lin, Chun-Hsien Hsu, Chung-Ze Wu, An-Tsz Hsieh, Chang-Hsun Hsieh, Yao-Jen Liang, Yen-Lin Chen, Dee Pei, Jin-Biou Chang

There are errors in the author affiliations. The affiliations should appear as shown here: Jiunn-Diann Lin^1,2^, Chun-Hsien Hsu^3^, Chung-Ze Wu^1,2^, An-Tsz Hsieh^1^, Chang-Hsun Hsieh^4^, Yao-Jen Liang^5^, Yen-Lin Chen^6^, Dee Pei^7^, Jin-Biou Chang^8^

1 Division of Endocrinology, Department of Internal Medicine, Shuang Ho Hospital, Taipei Medical University, New Taipei City, Taiwan, ROC, 2 Division of Endocrinology and Metabolism, Department of Internal Medicine, School of Medicine, College of Medicine, Taipei Medical University, Taipei, Taiwan, ROC, 3 Department of Family Medicine, Cardinal Tien Hospital, Fu Jen Catholic University School of Medicine, New Taipei City, Taiwan, ROC, 4 Division of Endocrinology and Metabolism, Department of Internal Medicine, Tri-Service General Hospital, National Defense Medical School, Taipei, Taiwan, ROC, 5 Department of Life Science, Fu Jen Catholic University, New Taipei City, Taiwan, ROC, 6 Department of Pathology, Cardinal Tien Hospital, Fu Jen Catholic University School of Medicine, New Taipei City, Taiwan, ROC, 7 Department of Internal Medicine, Cardinal Tien Hospital, Fu Jen Catholic University, New Taipei City, Taiwan, ROC, 8 Department of Pathology, National Defense Medical Center, Division of Clinical Pathology, Tri-Service General Hospital, Taipei, Taiwan, ROC.
